# United States Influenza Search Patterns Since the Emergence of COVID-19: Infodemiology Study

**DOI:** 10.2196/32364

**Published:** 2022-03-03

**Authors:** Owen Cai, Bernardo Sousa-Pinto

**Affiliations:** 1 Shadow Creek High School Pearland, TX United States; 2 Department of Community Medicine, Information and Health Decision Sciences Faculty of Medicine University of Porto Porto Portugal; 3 Center for Health Technologies and Services Research University of Porto Porto Portugal

**Keywords:** COVID-19, influenza, surveillance, media coverage, Google Trends, infodemiology, monitoring, trend, United States, information-seeking, online health information

## Abstract

**Background:**

The emergence and media coverage of COVID-19 may have affected influenza search patterns, possibly affecting influenza surveillance results using Google Trends.

**Objective:**

We aimed to investigate if the emergence of COVID-19 was associated with modifications in influenza search patterns in the United States.

**Methods:**

We retrieved US Google Trends data (relative number of searches for specified terms) for the topics influenza, Coronavirus disease 2019, and symptoms shared between influenza and COVID-19. We calculated the correlations between influenza and COVID-19 search data for a 1-year period after the first COVID-19 diagnosis in the United States (January 21, 2020 to January 20, 2021). We constructed a seasonal autoregressive integrated moving average model and compared predicted search volumes, using the 4 previous years, with Google Trends relative search volume data. We built a similar model for shared symptoms data. We also assessed correlations for the past 5 years between Google Trends influenza data, US Centers for Diseases Control and Prevention influenza-like illness data, and influenza media coverage data.

**Results:**

We observed a nonsignificant weak correlation (*ρ*= –0.171; *P*=0.23) between COVID-19 and influenza Google Trends data. Influenza search volumes for 2020-2021 distinctly deviated from values predicted by seasonal autoregressive integrated moving average models—for 6 weeks within the first 13 weeks after the first COVID-19 infection was confirmed in the United States, the observed volume of searches was higher than the upper bound of 95% confidence intervals for predicted values. Similar results were observed for shared symptoms with influenza and COVID-19 data. The correlation between Google Trends influenza data and CDC influenza-like-illness data decreased after the emergence of COVID-19 (2020-2021: *ρ*=0.643; 2019-2020: *ρ*=0.902), while the correlation between Google Trends influenza data and influenza media coverage volume remained stable (2020-2021: *ρ*=0.746; 2019-2020: *ρ*=0.707).

**Conclusions:**

Relevant differences were observed between predicted and observed influenza Google Trends data the year after the onset of the COVID-19 pandemic in the United States. Such differences are possibly due to media coverage, suggesting limitations to the use of Google Trends as a flu surveillance tool.

## Introduction

Google Trends is a tool that retrieves the relative amount of Google searches for specified terms based on location and a user-chosen timeframe [1]. Google Trends provides relative search volume data (on a scale of 0-100), consisting of the number of searches for specific terms relative to the total number of searches in the chosen timeframe [1]. Research based on Google Trends data is largely situated within the field of infodemiology, which is the practice of analyzing information in an electronic medium (particularly the internet) to improve public health and policy [2]. Information on the volume of web searches is a relatively new alternative to information gathered from traditional surveys, and several studies [3] have been conducted using search volume data since the first, which monitored the severe acute respiratory syndrome epidemic that occurred in 2002. The use of internet search data from Google Trends as a complement to traditional survey data is appealing, among other reasons, because data are provided on a real-time basis and web searches are performed anonymously, allowing for a greater range of data on sensitive topics [4-7].

Google Trends data have been used to monitor both chronic and acute diseases. Search volume data related to the common cold were found to be correlated with asthma incidence and to forecast asthma hospitalizations [8,9]. Additionally, Fang and colleagues [10] found that an increase in searches related to chronic obstructive pulmonary disease from 2007 to 2020 was correlated with several estimates of chronic obstructive pulmonary disease morbidity. For acute conditions, particularly infectious diseases, Seifter et al [11] noticed that Google searches on the keywords “Lyme disease, tick bite, [and] cough” reflected geographic locations and times of year that Lyme disease infections typically peak. Carneiro and Mylonakis [12] found that Google Trends search patterns for West Nile virus, respiratory syncytial virus, and avian influenza were correlated with those of seasonal or cyclical viral outbreaks. Yuan et al [13] found that searches on fever, gastroenteritis, and watery diarrhea were correlated with Google Trends norovirus data; some of these searches were also correlated with actual norovirus cases from New York, California, and the United States as a whole.

Several studies [14-17] have been conducted to assess internet search patterns on COVID-19 symptoms, individual protection equipment or measures, and vaccines, among others (although these were solely assessed the first months of the pandemic, when media coverage interest on the COVID-19 pandemic was particularly high [17]).

One of the most frequently assessed infectious diseases using Google Trends is influenza, which has been studied with mixed results. Cho et al [18] found there was a strong correlation between Korea Centers for Disease Control and Prevention (KCDC) influenza-like illness data and Google Trends data for 2007-2012 flu seasons [18]. Zhang et al [19] expanded upon the utility of Google Trends data by constructing an influenza outbreak predictor that was capable of successfully predicting influenza outbreaks. Similarly, Samaras et al [20] found a strong, statistically significant correlation between Google search data and influenza-like illness rates in Greece and in Italy, and using autoregressive integrated moving average models, they successfully predicted influenza peaks 4 weeks prior to their occurrence. On the other hand, although Ginsberg et al [4] found Google queries could be used to estimate influenza-like illness accurately in all 9 public health regions of the United States, they also noted potential artificial surges in influenza-related search volume after unusual media coverage that affected the ability of Google Trends data to be used in direct forecasting.

There also exists a substantial body of literature that examines the use of Google Flu Trends, which is an algorithm designed for the sole purpose of predicting influenza outbreaks [21,22]. Deployed in November 2008, Google Flu Trends used Google search data to estimate the intensity of an influenza epidemic and to predict US Centers for Disease Control and Prevention (CDC) data on the number of patient visits due to influenza-like illness. Its algorithm is based on the top 45 searches that had the highest correlation with US CDC influenza-like illness data (extracted from 50 billion of the most-searched Google terms) between 2003 and 2007; however, the 45 terms were never explicitly released, which means there is a lack of replicability. Furthermore, Google Flu Trends did not predict the 2009 influenza A–H1N1 pandemic [21,22] and overestimated the prevalence of flu cases for 100 out of 108 weeks starting in August 2011 [22]. The underestimation of the first 2009 wave of H1N1 in the United States was partially attributed [23] to the public’s general lack of knowledge regarding H1N1 (in contrast with the second wave, from 2009-2010, which reflected actual flu patterns). Google Flu Trends was unsuccessful in its attempts to monitor and predict the course of influenza outbreaks solely based on internet search data, which serves as a warning of the volatility of internet search data and its potential to not reflect true disease case data.

Success in monitoring or predicting outbreaks using Google Trends data depends on the keywords used. In Google Trends data collection, selecting the proper keywords is “key for valid results [6].” Kang et al [24] found that based on the specific keyword used (“influenza a,” “fever,” “cold,” or “cough”), the correlation between Google Trends and influenza surveillance data (from 56 sentinel clinics of the official Guangdong CDC from 2008 to 2011) would change—for all 4 years, “fever” was significantly correlated with Guangdong CDC data; however, “H1N1” was not significantly correlated with any year’s data. Ultimately, Kang et al [24] suggest that analysts should be cautious when there is high media coverage for a particular influenza season or strain, because of the potential bias in internet search patterns.

Similarly, however, the emergence of COVID-19 could have also distorted Google Trends influenza search patterns. Both COVID-19 and influenza are respiratory diseases and share several common symptoms (such as fever, cough, and sore throat [25]) alongside seasonality [26]. An analogous scenario was demonstrated [27], identifying that searches for asthma and chronic obstructive pulmonary disease peaked in March 2020, and the increase in asthma searches was attributed to the potential shared respiratory effects of COVID-19 and asthma and to the large media coverage on COVID-19.

The severity of the COVID-19 pandemic has led to a heightened sense of risk and constant media coverage, which has caused individuals to search the internet for more information on COVID-19. Because surges in COVID-19 searches could affect Google Trends flu search patterns, altering Google Trends’ capacity to be used as a supplemental surveillance tool, we aimed to assess and quantify the extent to which the emergence of COVID-19 was associated with fluctuations in Google Trends influenza search patterns in the United States.

## Methods

### Study Design

We collected Google Trends data for influenza, COVID-19, and their shared symptoms using the framework by Mavragani and Ochoa [6]. We (1) determined the correlation between influenza searches and COVID-19 searches during the first year of the COVID-19 pandemic in the United States (January 21, 2020 to January 20, 2021), (2) developed a time series model based on data from previous years to predict flu search data which we compared with observed data in order to detect irregularities in influenza search patterns since the emergence of COVID-19; (3) developed a time series models based on data from previous years to predict shared symptoms data which we compared with observed data to detect irregularities in shared symptoms search patterns since the emergence of COVID-19; and (4) determined the correlations between search data and data from other sources for the past 5 years (including US CDC surveillance data and influenza media coverage volume) in order to detect any changes since the emergence of COVID-19.

### Data Collection

#### Keyword Selection

Although in past Google Trends flu research [18,20,24], specific keywords have been used, we employed search topics, which are a group of terms that share the same concept across languages. Topics cover an array of variations, typos, and related searches, precluding the need to enter a set of individual terms, while maintaining the consistency of search queries across all timeframes. We extracted Google Trends data using the topics “Coronavirus disease 2019” and “Influenza”, and queried cough + fever + “sore throat” + “difficulty breathing” for assessing the shared symptoms between COVID-19 and influenza; specific categories and subcategories within Google Trends were not selected for any keyword searches.

#### Region and Period Selection

We retrieved Google Trends data for the United States at a national level. In addition, we extracted data for the 4 most populous states (California, Texas, Florida, and New York) to assess regional variations in the strength of correlations and predictions. We extracted data from January 21, 2016 to January 20, 2021, corresponding to a period of 5 complete years. Each full year was defined as starting on January 21, because the US CDC confirmed the first COVID-19 infection in the United States on January 21, 2020. This allowed us to analyze a full year after the first COVID-19 case and streamline the collection of past years’ data. For simplicity, we will refer to each period set using the years (ie, for data extracted from January 21, 2016 to January 20, 2017, we will simply state *2016-2017*).

#### Other Data Sources

The US CDC monitors the cyclical progression of influenza by tracking weekly cases of influenza-like illness (defined as a fever, cough, or sore throat without known cause other than the flu) [25]. We retrieved these data from January 21, 2016 to January 20, 2021, for the United States data and for California, Texas, and New York (no data were available for Florida) from the CDC’s FluView Interactive App [28]. Since the FluView shows data from week 40 of one year to week 39 of the next, we spliced together the influenza-like illness data from different flu seasons.

We accessed an open-source platform (Media Cloud) to retrieve the percentage of media stories concerning influenza. We extracted US data from January 21, 2016 to January 20, 2021 using the query “flu OR influenza.” Data from each of the 4 most populous states were also retrieved. Weekly averages were calculated based on daily data.

### Data Analysis

Data analysis was performed using SPSS (version 25; IBM Corp) and R (version 4.0.4) software. *P* values<.05 were considered statistically significant.

Spearman correlation coefficients were calculated for the entire year and quarterly periods (13 weeks) of the year to assess the relationship between COVID-19 and influenza data from Google Trends.

We then assessed how predicted Google Trends flu data differed from actual data since the emergence of COVID-19 to detect eventual irregularities in flu search patterns. To do that, we extracted Google Trends flu data from 2016-2021 and, based on data from 2016-2020. We built seasonal autoregressive integrated moving average (SARIMA) models [27]. An identical process was performed to compare forecasted and observed Google Trends data for shared symptoms relative search volume.

SARIMA models were used to forecast 2020-2021 data based on past data provided and accounting for seasonal patterns. The models were defined by (*p*,*d*,*q*)(*P’*,*D*,*Q*)*s*, with *p* corresponding to the order of autoregression, *d* corresponding to the degree of difference, *q* corresponding to the order of the moving average part, *P’* corresponding to the seasonal order of autoregression, *D* corresponding to the degree of difference following seasonal integration, *Q* corresponding to the seasonal moving average, and *s* corresponding to the length of the seasonal period. We set *s*=52 weeks (since there are approximately 52 weeks in a year), and we selected *d* and *D* so that the 2016-2020 time series appeared stationary (ie, with a constant variance and no extreme fluctuations or overall increasing or decreasing behavior); *p* and *P’* were selected based on partial autocorrelation function plots, and *q* and *Q* were selected based on autocorrelation function plots. SARIMA models were selected based on the results of the Ljung-Box test and on the Akaike information criteria of tested models.

To compare predicted and observed relative search volumes, we calculated the Spearman correlation coefficients for 2020-2021 and for each quarter. We calculated the mean absolute difference and percent difference between observed and predicted Google Trends data and determined the number of weeks for which the observed data exceeded predicted confidence intervals.

We calculated the Spearman correlation coefficients between Google Trends data for influenza and US CDC influenza-like illness data and between Google Trends data for influenza and Media Cloud influenza media coverage data from 2016 to 2021. To assess whether CDC influenza-like illness and media coverage data differed substantially in 2020-2021 compared to those from previous years, we built SARIMA models and determined the number of weeks for which the observed data exceeded predicted confidence intervals (Multimedia Appendix 1).

## Results

### Google Trends COVID-19 and Influenza Data

**Figure 1:**
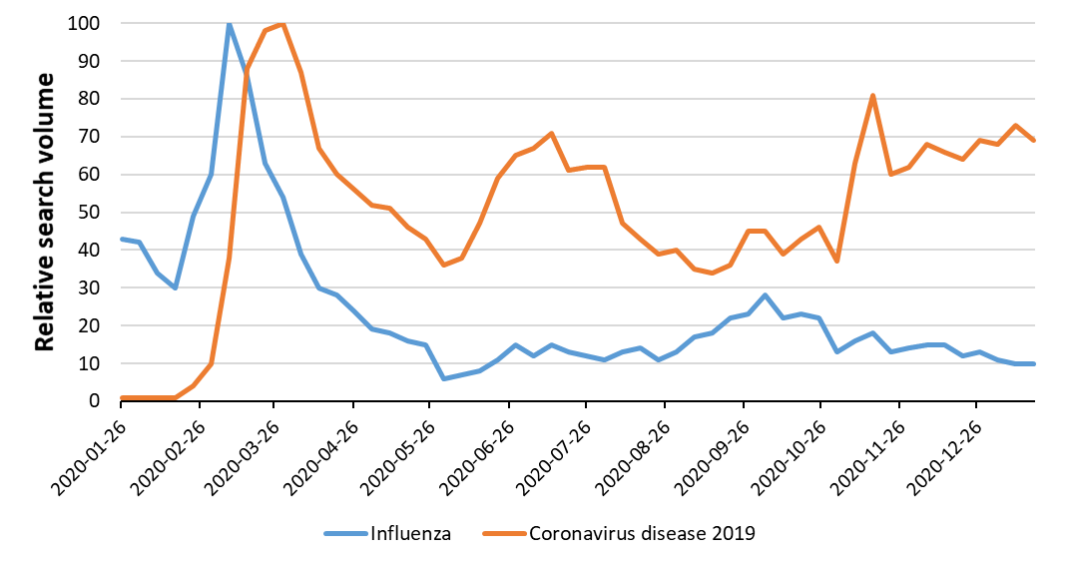
Influenza and COVID-19 topic relative search volumes from January 21, 2020 to January 20, 2021.

**Figure 2:**
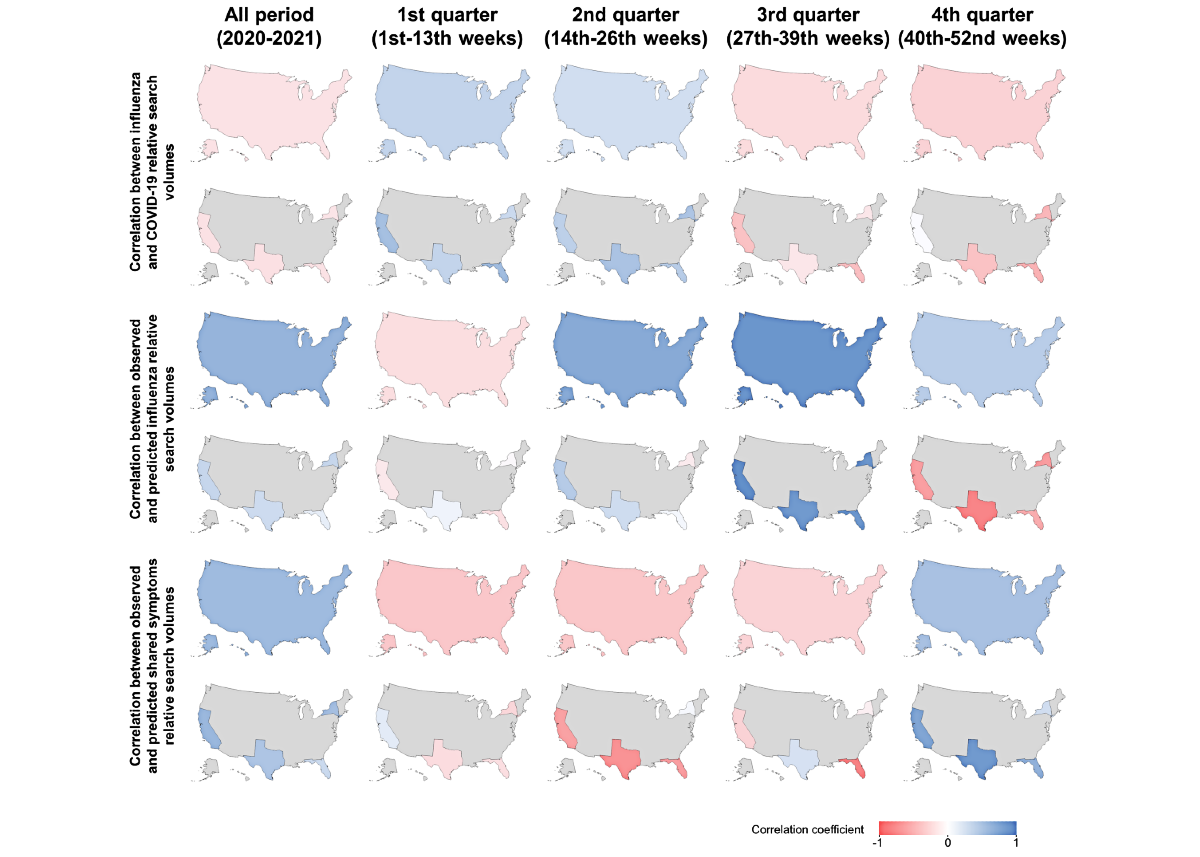
Correlation heatmaps (national and statewide) for (A) influenza and COVID-19 relative search volumes, (B) observed and predicted influenza relative search volumes, and (C) observed and predicted shared symptoms relative search volumes.

We observed nonsignificant weak correlations between influenza and COVID-19 Google Trends data for the United States at the national level (*ρ*=–0.171; *P*=.23) and for each state (California: *ρ*=–0.179; *P*=.20; Florida: *ρ*=–0.173; *P*=.22; New York: *ρ*=–0.161; *P*=.26; Texas: *ρ*=–0.188; *P*=.18) (Figure 1). Similarly, quarterly correlations were nonsignificant (Table 1) and mostly weak (Figure 2).

**Table 1:** Correlation between influenza and COVID-19 relative search volumes.

Region and period^a^					*ρ*			*P* value
**United States of America**								
	**Entire period**			–0.171			.23	
		1st quarter	0.358			.23		
		2nd quarter	0.271			.37		
		3rd quarter	–0.224			.46		
		4th quarter	–0.281			.35		
**California**								
	**Entire period**			–0.179			.20	
		1st quarter	0.498			.08		
		2nd quarter	0.391			.19		
		3rd quarter	–0.392			.19		
		4th quarter	0.012			.97		
**Florida**								
	**Entire period**			–0.173			.22	
		1st quarter	0.531			.06		
		2nd quarter	0.409			.16		
		3rd quarter	–0.405			.17		
		4th quarter	–0.482			.10		
**New York**								
	**Entire period**			–0.161			.26	
		1st quarter	0.311			.30		
		2nd quarter	0.487			.09		
		3rd quarter	–0.146			.63		
		4th quarter	–0.465			.11		
**Texas**								
	**Entire period**			–0.188			.18	
		1st quarter	0.354			.24		
		2nd quarter	0.503			.08		
		3rd quarter	–0.144			.64		
		4th quarter	–0.392			.18		

^a^Entire period: January 21, 2020 to January 20, 2021; 1st quarter: January 26, 2020 to April 19, 2020; 2nd quarter: April 26, 2020 to July 19, 2020; 3rd quarter: July 26, 2020 to October 18, 2020; 4th quarter: October 25, 2020 to January 17, 2021.

### Predicted Versus Observed Google Trends Influenza Data

**Figure 3:**
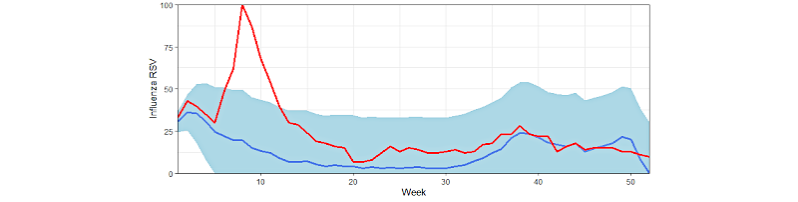
Predicted and observed influenza relative search volume (RSV) values from January 21, 2020 to January 20, 2021. The red line shows the observed relative search values for influenza, the blue line represents the predicted values for influenza searches, and the shaded blue area represents the confidence intervals for predicted values.

At the national level, observed influenza relative search volume fell outside predicted confidence intervals for 6 out of 52 weeks (11.5%) (Figure 3), all of which occurred during the first quarter. The average difference between observed and predicted relative search volume values was 12.9 units (mean percent difference 48.4%). For the entire period, the correlation between observed and predicted relative search volume, *ρ*=0.632 (*P*<.001) (Figure 2); however, for the first quarter, the correlation (which included the 6 weeks for which observed Google Trends values went beyond the confidence interval for predicted values) reported value was strikingly different and not significant (*ρ*=–0.204; *P*=.28) (Table 2).

For California, Florida, and Texas, all weeks in which observed influenza relative search volumes fell outside predicted confidence intervals occurred during the first quarter.

**Table 2:** Correlation between predicted and observed influenza relative search volumes.

Region and period^a^					*ρ*			*P* value			Weeks outside predicted CIs, n (%^b^)
**United States of America**											
	**Entire period**			0.632			<.001			6 (11.5)	
		1st quarter	–0.204			.28			6 (46.2)		
		2nd quarter	0.720			.02			0 (0)		
		3rd quarter	0.899			<.001			0 (0)		
		4th quarter	0.417			.002			0 (0)		
**California**											
	**Entire period**			0.338			.01			7 (13.5)	
		1st quarter	–0.132			.67			7 (53.8)		
		2nd quarter	0.436			.14			0 (0)		
		3rd quarter	0.946			<.001			0 (0)		
		4th quarter	–0.626			.02			0 (0)		
**Florida**											
	**Entire period**			0.130			.36			10 (19.2)	
		1st quarter	–0.184			.55			10 (76.9)		
		2nd quarter	0.050			.87			0 (0)		
		3rd quarter	0.806			<.001			0 (0)		
		4th quarter	–0.514			.07			0 (0)		
**New York**											
	**Entire period**			0.338			.01			21 (40.4)	
		1st quarter	–0.022			.94			10 (76.9)		
		2nd quarter	–0.114			.71			4 (30.8)		
		3rd quarter	0.866			<.001			2 (15.4)		
		4th quarter	–0.634			.02			5 (38.5)		
**Texas**											
	**Entire period**			0.292			.04			5 (9.6)	
		1st quarter	0.082			.79			5 (38.5)		
		2nd quarter	0.288			.34			0 (0)		
		3rd quarter	0.861			<.001			0 (0)		
		4th quarter	–0.804			<.001			0 (0)		

^a^Entire period: January 21, 2020 to January 20, 2021; 1st quarter: January 26, 2020 to April 19, 2020; 2nd quarter: April 26, 2020 to July 19, 2020; 3rd quarter: July 26, 2020 to October 18, 2020; 4th quarter: October 25, 2020 to January 17, 2021.

^b^For the entire period, out of 52 weeks. For a quarter, out of 13 weeks.

### Predicted Versus Observed Google Trends Data on Shared Symptoms

**Figure 4:**
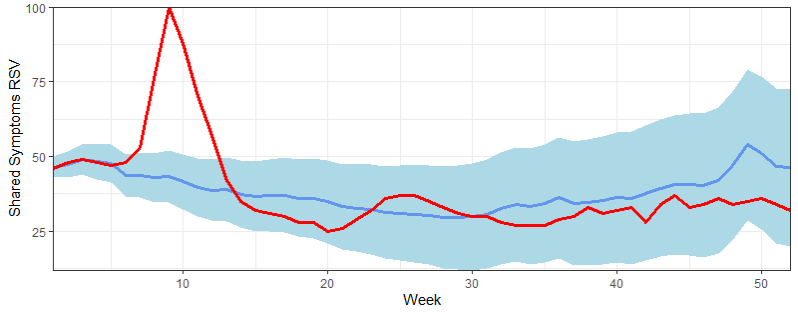
Predicted and actually observed relative search volume (RSV) values on symptoms common to both influenza and COVID-19 from January 21, 2020 to January 20, 2021. The red line shows the observed relative search values, the blue line represents the predicted relative search values, and the shaded blue area represents the confidence intervals for predicted values.

At the national level, observed relative search volume data for shared symptoms fell outside predicted confidence intervals (Figure 4) for the same 6 weeks as those of influenza relative search volume data. The average difference in relative search volume between the actual and predicted data was 8.7 units (mean percent difference 20.2%). The correlation for the entire period was significant (*ρ*=0.578; *P*<.001) (Table 3). For individual states, a more diverse pattern was observed when comparing observed versus predicted shared symptoms relative search volume data (e.g., in California, there were only 4 weeks outside predicted intervals – all occurring during the first quarter -, while in New York, there were 18 weeks outside predicted intervals, occurring in all quarters) (Figure 2).

**Table 3:** Correlations between predicted and actually observed shared symptoms (between influenza and COVID-19) relative search volumes.

Region and period^a^					*ρ*			*P* value			Weeks outside predicted CIs, n (%^b^)
**United States of America**											
	**Entire period**			0.578			<.001			6 (11.5)	
		1st quarter	–0.354			.24			6 (46.2)		
		2nd quarter	–0.359			.23			0 (0)		
		3rd quarter	–0.273			.37			0 (0)		
		4th quarter	0.518			.07			0 (0)		
**California**											
	**Entire period**			0.603			<.001			4 (7.7)	
		1st quarter	0.155			.61			4 (30.8)		
		2nd quarter	–0.610			.03			0 (0)		
		3rd quarter	–0.281			.35			0 (0)		
		4th quarter	0.759			.003			0 (0)		
**Florida**											
	**Entire period**			0.303			.03			9 (17.3)	
		1st quarter	–0.200			.52			8 (61.5)		
		2nd quarter	–0.599			.03			0 (0)		
		3rd quarter	–0.768			.002			0 (0)		
		4th quarter	0.615			.03			1 (7.7)		
**New York**											
	**Entire period**			0.537			<.001			18 (34.6)	
		1st quarter	–0.254			.40			7 (53.8)		
		2nd quarter	0.041			.89			3 (23.1)		
		3rd quarter	–0.083			.79			1 (7.7)		
		4th quarter	0.274			.36			7 (53.8)		
**Texas**											
	**Entire period**			0.484			<.001			21 (40.4)	
		1st quarter	–0.214			.48			6 (46.2)		
		2nd quarter	–0.711			.006			5 (38.5)		
		3rd quarter	0.237			.44			0 (0)		
		4th quarter	0.864			<.001			10 (76.9)		

^a^Entire period: January 21, 2020 to January 20, 2021; 1st quarter: January 26, 2020 to April 19, 2020; 2nd quarter: April 26, 2020 to July 19, 2020; 3rd quarter: July 26, 2020 to October 18, 2020; 4th quarter: October 25, 2020 to January 17, 2021.

^b^For the entire period, out of 52 weeks. For a quarter, out of 13 weeks.

### Correlations Between Google Trends and Other Data Sources

**Figure 5:**
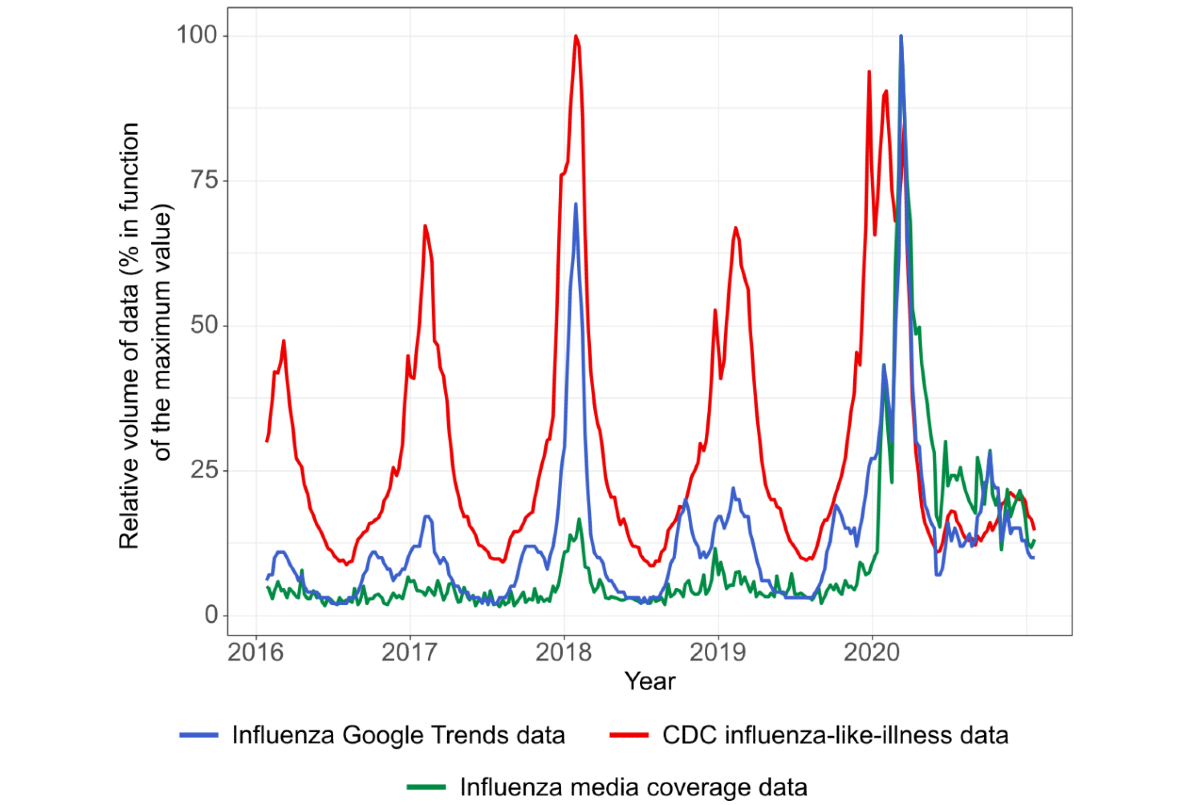
Relative volume of data for influenza Google Trends data, CDC influenza-like illness data, and influenza media coverage data.

For each of the 4 previous years, strong positive correlations were observed in the United States for CDC influenza-like illness data and Google Trends relative search volume data (Figure 5) and for CDC influenza-like illness data and media coverage; correlations for 2020-2021 were weaker than those of the previous years. Similarly, for 2020-2021, correlations between CDC influenza-like illness data and media coverage for influenza in each state, except New York, were weaker than for those of the previous years. Correlations between Google Trends influenza and influenza media coverage tended to be as strong in 2020-2021 as in the previous years (Table 4).

For CDC influenza-like illness data, there was a strong correlation between observed and predicted data (*ρ*=0.701; *P*<.001). Despite this finding, on average, observed values tended to be lower than forecasted values but were still within forecasted confidence intervals when considering the entire 2020-2021 period. For influenza media coverage, the correlation between observed and predicted values was low (*ρ*=–0.063; *P*=.66), with observed data falling outside predicted confidence intervals for 14 weeks, mostly during the first quarter.

**Table 4:** Correlations between Google Trends relative search volume, US Centers for Disease Control and Prevention influenza-like illness case report, and media coverage data.

Region and period^a^			Relative search volume and case reports				Relative search volume and media coverage					Relative case reports and media coverage		
			*ρ*		*P* value		*ρ*		*P* value		*ρ*			*P* value
**United States of America**														
	2016-2017	0.753		<.001		0.483		<.001		0.643			<.001	
	2017-2018	0.869		<.001		0.607		<.001		0.689			<.001	
	2018-2019	0.846		<.001		0.878		<.001		0.864			<.001	
	2019-2020	0.902		<.001		0.707		<.001		0.720			<.001	
	2020-2021	0.643		<.001		0.746		<.001		0.440			.001	
**California**														
	2016-2017	0.739		<.001		0.483		<.001		0.586			<.001	
	2017-2018	0.817		<.001		0.648		<.001		0.740			<.001	
	2018-2019	0.733		<.001		0.805		<.001		0.700			<.001	
	2019-2020	0.744		<.001		0.604		<.001		0.668			<.001	
	2020-2021	0.408		.002		0.706		<.001		0.093			.51	
**Florida**														
	2016-2017	—^b^		—		0.195		.17		—			—	
	2017-2018	—		—		0.571		<.001		—			—	
	2018-2019	—		—		0.733		<.001		—			—	
	2019-2020	—		—		0.521		<.001		—			—	
	2020-2021	—		—		0.694		<.001		—			—	
**New York**														
	2016-2017	0.837		<.001		0.511		<.001		0.518			<.001	
	2017-2018	0.766		<.001		0.668		<.001		0.484			<.001	
	2018-2019	0.867		<.001		0.815		<.001		0.726			<.001	
	2019-2020	0.826		<.001		0.684		<.001		0.654			<.001	
	2020-2021	0.685		<.001		0.825		<.001		0.533			<.001	
**Texas**														
	2016-2017	0.671		<.001		0.379		.006		0.464			<.001	
	2017-2018	0.882		<.001		0.519		<.001		0.531			<.001	
	2018-2019	0.868		<.001		0.546		<.001		0.519			<.001	
	2019-2020	0.919		<.001		0.495		<.001		0.543			<.001	
	2020-2021	0.449		<.001		0.707		<.001		0.184			.19	

^a^Each period is defined as starting in January 21 and ending in January 20 of the subsequent year.

^b^There were no influenza-like illness data for Florida.

## Discussion

### Principal Results

In this study, we noted atypical Google Trends influenza search patterns in the year after the emergence of COVID-19 compared to expected patterns, which could limit the accuracy when using Google Trends as an influenza surveillance tool. This claim is supported by (1) disparities between the predicted and observed influenza relative search volume data, (2) a lack of significant correlations in the first quarter between forecasted and observed relative search volume for influenza data, and (3) weak correlations between CDC influenza-like illness and Google Trends influenza data.

For the United States as a whole, in 6 out of 52 weeks, influenza relative search volumes exceed predicted confidence intervals. Similar results were observed in each assessed state. Importantly, weeks when observed values exceeded predicted ranges for the United States (and most weeks for individual states) were within the first quarter, which was also when correlations between actual and predicted Google Trends influenza data were weaker than 2016-2020 correlations. Interestingly, this was also the quarter in which influenza media coverage was at its highest.

The strength of the correlations between CDC influenza-like illness and Google Trends influenza data decreased in 2020-2021 compared with those of previous years. The same, however, did not occur for the correlations between influenza media coverage and Google Trends influenza data, which remained strong, even given the increase in influenza media coverage in the first quarter of 2020-2021. These findings support the connection between Google Trends searches and media coverage on influenza, which have remained closely associated throughout the pandemic.

We did not observe a strong positive correlation between Google Trends data for COVID-19 and influenza from January 21, 2020 to January 20, 2021 in states or the country as a whole. While our study does not establish a direct correlation or causal link between the 2 diseases, the timing of the search peak of influenza and of shared symptoms (Figures 3 and 4) supports the hypothesis that high interest in COVID-19, its symptoms, and main differential diagnoses (including the flu) during the first quarter may have prompted higher volumes of news discussing influenza, which likely affected the search patterns for the flu (similar search peaks have been observed for other respiratory diseases, such as asthma and chronic obstructive pulmonary disease, possibly for the same reasons [27]). During this quarter (from January 19 to April 12), the first diagnosis of COVID-19 (on January 21) and a declaration of a public health emergency (February 3) occurred in the United States [29]. In fact, peaks in relative search volumes for influenza (in the week of March 8, 2020) and COVID-19 (in the week of March 29) occurred in quick succession (Figure 1). Furthermore, during the week that influenza relative search volume peaked, the World Health Organization declared COVID-19 a pandemic (on March 11) and the United States declared a national emergency (on March 13) [29].

### Comparison With Prior Work

Our study highlights that flu searches can reflect not only the epidemiology of influenza but also be influenced by external factors, specifically media-garnering developments such as the COVID-19 pandemic, which provides evidence to counter claims that Google Trends can be used single handedly to predict influenza outbreaks accurately [19,20]. When we compared forecasted and observed flu search volume data, 6 weeks of forecasts were inaccurate, which included the week in which flu relative search volume peaked, because the peak in relative search volume for influenza and spikes in search interest were neither reflected in CDC influenza-like illness case report data nor predicted by the SARIMA model. Such spikes are often the least predictable due to rapid media propagation, highlighting possible limitations of predictive models in accounting for sudden surges in searches. Previous studies [24] had already warned of the possibility that high-media events inflate influenza searches and distort flu search patterns. We investigated COVID-19 and noticed closely timed relative search volume peaks for influenza and COVID-19, with some unpredicted observed influenza relative search volume activity in the same timeframe that COVID-19 relative search volume and media presence rose [24].

Previous works have also assessed the influence of media coverage on internet search activity [17,27,30]. This influence does not include solely infectious diseases. For example, Cervellin et al [23] found that searches for the keyword “autism” surged consistently in May, potentially due to World Autism Day in April, but were likely not in accordance with real epidemiological data. In our study, Google Trends influenza data, similar to those for asthma and chronic obstructive pulmonary disease [27], peaked in March 2020, in the same week that the United States declared a national emergency.

Importantly, other than for 2020-2021, Google Trends and CDC influenza-like illness data displayed strong correlations, in accordance with the findings of a previous study [18], which showed strong correlations between national influenza surveillance data and Google Trends influenza data in Korea for the 2007-2012 flu seasons. Although our results suggest that Google Trends cannot be used as a sole tool to predict influenza outbreaks, they do not preclude the use of Google Trends as a tool for predicting the present and the very near future that is complementary to traditional surveillance systems, which has been previously discussed [31,32]. Using Google Trends data in combination with past influenza data may help decreasing the error of flu surveillance and hospitalizations predictions in the United States in comparison with using only past surveillance or hospitalization data [33,34]. The application of data correction methods to Google Trends may be particularly useful in improving the accuracy of predictions [32,35].

We were also able to quantify media coverage on influenza. While previous Google Trends influenza-like illness and influenza research assessed correlations between Google Trends data and official surveillance data with yearly intervals of data [18,24], when appropriate, we also used quarterly data. There were relevant across-quarter differences both in terms of correlations between observed and predicted data and in the number of weeks that the observed data went beyond predicted intervals. Using smaller intervals of time addresses seasonality and significant events. In fact, the first quarter of 2020-2021 included many of the first declarations of COVID-19 and emergency declarations, while in the third quarter, influenza searches returned to a relatively predictable pattern. This may have been, in part, because of the summer season in the Northern Hemisphere, which is a period of low activity for both seasonal coronaviruses and influenza [30].

### Limitations

This study has some limitations. First, the specific keywords that Google uses for defining the influenza and COVID-19 disease topics are not directly stated, but using topic searches was preferable to using search terms because topics encompass a wide range of relevant keywords.

For shared symptoms, we were not able to use topics because there was no topic encompassing all symptoms, and queries had to be built with combinations of keywords. The choice of keywords can decisively influence results [24]. In our study, there were more potentially relevant terms than those we included, but many of these symptoms tended to be broad, thus, to minimize the effect of broad search terms, we limited the number of terms in our search query. Even with this concession, at both the nation and state level, there were noticeable variations in observed and predicted shared symptoms relative search volume, which limited drawing conclusions based on shared symptoms data.

Another limitation is the fact that Google Trends presents searches in relative volume instead of as an absolute number of searches. The latter would facilitate comparisons between influenza and COVID-19 queries and reveal more information about the absolute search interest in each disease. Additionally, because Google Trends is based on Google search engine data, older individuals, individuals with less education, individuals with low income, and individuals in rural areas or isolated from technology may be underrepresented in internet searches [2].

The weaker correlation between CDC influenza-like illness and Google Trends influenza data for 2020-2021 may, not only be explained by changes in search patterns for influenza, but also, by a decrease in actual case numbers for influenza after the emergence of COVID-19 (eg, from the widespread adoption of individual protective measures) [36], which may also hamper the reliability of using Google Trends in influenza surveillance. However, in the first quarter of 2020-2021, when we detected the greatest differences in Google Trends influenza patterns, there was no observable decline in CDC influenza-like illness data compared with that expected based on previous years’ data.

Finally, we only used data from a single country; conclusions may not be generalizable to other countries; however, we conducted an exploratory analysis, applying the same methodology to other countries with English as one of the official languages (such as Canada, United Kingdom, Ireland, Australia, and New Zealand) and displaying high-quality relative search volume data, which demonstrated consistent findings for correlations between predicted and observed Google Trends influenza data, with more disparate results observed for shared symptoms (Multimedia Appendices 2 and 3). Despite the focus on the United States, the methodology framework of our study can be extended to other countries with developed national influenza surveillance systems and reliable access to the internet, which would provide a new understanding of national-scale variations in influenza search patterns since the onset of COVID-19.

This study also has important strengths. We were able to compare observed data and predicted data by using time series forecasting methods for influenza data and shared symptoms data. We did not build models simultaneously incorporating Google Trends and CDC influenza-like illness data, as suggested by some [37], because our aim was, not to nowcast influenza-like illness rates, but rather, to assess correlations and differences between observed and predicted values). In addition, we assessed correlations between Google Trends influenza and CDC influenza-like illness data for a 5-year period, finding evidence that there may be ramifications from the emergence of COVID-19 on US disease surveillance. 

In future studies, as the COVID-19 pandemic in the United States (and all states) constantly evolves due to new variants and waves of infections, research into Google Trends influenza searches after January 2021 would help to continually assess the shifts in Google Trends searches and the reliability of Google Trends. Each subnational territory’s Google Trends influenza and COVID-19 relative search volume data could yield a more comprehensive picture of regional search patterns.

### Conclusions

Influenza search patterns deviated from those of previous years once COVID-19 gained media presence, even when accounting for the seasonality of influenza searches, and 2020-2021 yielded the weakest correlations between CDC and Google Trends flu data over a 5-year period—both findings suggest that the accuracy of Google Trends as a supplementary influenza surveillance tool in periods of highly mediatized respiratory infections breakouts should be carefully assessed. Furthermore, although we cannot posit that COVID-19 search interest directly influences Google Trends flu data, we found that media coverage likely factored into the noticeably irregular influenza search patterns, and we caution against solely relying on Google Trends data for influenza surveillance, because media influence may cause Google Trends searches to diverge from normal patterns.

## Appendix

Multimedia Appendix 1 Seasonal autoregressive integrated moving average (SARIMA) models for forecasting influenza and shared symptoms relative search volume data for 2020-2021.

Multimedia Appendix 2 Correlation between influenza and COVID-19 in majority native English-speaking countries other than the United States of America.

Multimedia Appendix 3 Correlations between predicted and observed (1) influenza relative search values and (2) shared influenza and COVID-19 symptoms relative search values in majority native English-speaking countries other than the United States.

## Abbreviations

CDC: US Centers for Disease Control and Prevention
KCDC: Korea Centers for Disease Control and Prevention
SARIMA: seasonal autoregressive integrated moving average
