# Supplementary material for: United States Influenza Search Patterns Since the Emergence of COVID-19: Infodemiology Study
Source: JMIR Public Health Surveill. 2022 Mar 3;8(3):e32364. doi: 10.2196/32364 (PMC8896565; doi:10.2196/32364)
Supplement: Multimedia Appendix 1 [file publichealth_v8i3e32364_app1.docx]

**Supplementary Table 1**: Seasonal autoregressive integrated moving average (SARIMA) models for forecasting influenza and shared symptoms relative search volume data for 2020-2021

| **Scope of the model** | **SARIMA final model** |
| --- | --- |
| Influenza relative search volume data forecast |  |
| United States of America | (2,0,4), (1,2,0)_52_ |
| California | (1,1,2), (1,1,1)_52_ |
| Florida | (1,1,1), (1,1,2)_52_ |
| New York | (1,1,2), (2,1,2)_52_ |
| Texas | (2,1,2), (1,1,2)_52_ |
| Shared symptoms relative search volume data forecast |  |
| United States of America | (2,2,2), (1,1,1)_52_ |
| California | (2,1,1), (1,1,1)_52_ |
| Florida | (1,1,1), (1,1,2)_52_ |
| New York | (1,1,1), (1,1,1)_52_ |
| Texas | (2,1,1), (1,1,1)_52_ |
| CDC Influenza-like Illness data forecast | (1,1,2), (1,1,1)_52_ |
| Influenza media coverage data forecast | (1,1,0), (1,1,1)_52_ |
