# Supplementary material for: United States Influenza Search Patterns Since the Emergence of COVID-19: Infodemiology Study
Source: JMIR Public Health Surveill. 2022 Mar 3;8(3):e32364. doi: 10.2196/32364 (PMC8896565; doi:10.2196/32364)
Supplement: Multimedia Appendix 2 [file publichealth_v8i3e32364_app2.docx]

**Supplementary Table 2.** Correlation between Influenza and COVID-19 in majority native English-speaking countries other than the United States of America

|  | Spearman correlation coefficient | *P* value |
| --- | --- | --- |
| Australia |  |  |
| Entire Period (January 21, 2020 to January 20, 2021) | 0.203 | .15 |
| 1st Quarter (1st-13th weeks) | 0.880 | <.001 |
| 2nd Quarter (14th-26th weeks) | 0.564 | .04 |
| 3rd Quarter (27th-39th weeks) | 0.894 | <.001 |
| 4th Quarter (40th-52nd weeks) | 0.735 | .004 |
| Canada |  |  |
| Entire Period (January 21, 2020 to January 20, 2021) | 0.418 | .002 |
| 1st Quarter (1st-13th weeks) | 0.624 | .02 |
| 2nd Quarter (14th-26th weeks) | 0.890 | <.001 |
| 3rd Quarter (27th-39th weeks) | 0.764 | .002 |
| 4th Quarter (40th-52nd weeks) | -0.001 | .99 |
| Ireland |  |  |
| Entire Period (January 21, 2020 to January 20, 2021) | 0.302 | .03 |
| 1st Quarter (1st-13th weeks) | 0.472 | .10 |
| 2nd Quarter (14th-26th weeks) | 0.553 | .05 |
| 3rd Quarter (27th-39th weeks) | 0.798 | .001 |
| 4th Quarter (40th-52nd weeks) | -0.120 | .70 |
| New Zealand |  |  |
| Entire Period (January 21, 2020 to January 20, 2021) | 0.440 | .001 |
| 1st Quarter (1st-13th weeks) | 0.766 | .002 |
| 2nd Quarter (14th-26th weeks) | 0.906 | <.001 |
| 3rd Quarter (27th-39th weeks) | 0.565 | .04 |
| 4th Quarter (40th-52nd weeks) | 0.040 | .90 |
| United Kingdom |  |  |
| Entire Period (January 21, 2020 to January 20, 2021) | 0.364 | .008 |
| 1st Quarter (1st-13th weeks) | 0.614 | .03 |
| 2nd Quarter (14th-26th weeks) | 0.650 | .02 |
| 3rd Quarter (27th-39th weeks) | 0.742 | .004 |
| 4th Quarter (40th-52nd weeks) | -0.710 | .007 |
