# Supplementary material for: United States Influenza Search Patterns Since the Emergence of COVID-19: Infodemiology Study
Source: JMIR Public Health Surveill. 2022 Mar 3;8(3):e32364. doi: 10.2196/32364 (PMC8896565; doi:10.2196/32364)
Supplement: Multimedia Appendix 3 [file publichealth_v8i3e32364_app3.docx]

**Supplementary Table 3.** Correlation between predicted and actually observed (i) influenza relative search values, and (ii) shared symptoms (between influenza and COVID-19) relative search values in majority native English-speaking countries other than the United States of America.

|  | Spearman correlation coefficient (*P*-value) | *N* (%) weeks outside predicted confidence intervals |
| --- | --- | --- |
| **A. Influenza relative search values** |  |  |
| Australia |  |  |
| Entire Period (January 21, 2020 to January 20, 2021) | 0.611 (<.001) | 8 (15.4) |
| 1st Quarter (1st-13th weeks) | 0.593 (.04) | 8 (61.5) |
| 2nd Quarter (14th-26th weeks) | 0.414 (.16) | 0 |
| 3rd Quarter (27th-39th weeks) | 0.930 (<.001) | 0 |
| 4th Quarter (40th-52nd weeks) | 0.144 (.64) | 0 |
| Canada |  |  |
| Entire Period (January 21, 2020 to January 20, 2021) | 0.597 (<.001) | 11 (21.2) |
| 1st Quarter (1st-13th weeks) | -0.192 (.59) | 9 (69.2) |
| 2nd Quarter (14th-26th weeks) | 0.584 (.04) | 0 |
| 3rd Quarter (27th-39th weeks) | 0.932 (<.001) | 2 (15.4) |
| 4th Quarter (40th-52nd weeks) | 0.047 (.88) | 0 |
| Ireland |  |  |
| Entire Period (January 21, 2020 to January 20, 2021) | 0.521 (<.001) | 5 (9.6) |
| 1st Quarter (1st-13th weeks) | -0.072 (.82) | 5 (38.5) |
| 2nd Quarter (14th-26th weeks) | 0.618 (.02) | 0 |
| 3rd Quarter (27th-39th weeks) | 0.960 (<.001) | 0 |
| 4th Quarter (40th-52nd weeks) | -0.177 (.56) | 0 |
| New Zealand |  |  |
| Entire Period (January 21, 2020 to January 20, 2021) | 0.556 (<.001) | 11 (21.2) |
| 1st Quarter (1st-13th weeks) | 0.462 (.11) | 10 (76.9) |
| 2nd Quarter (14th-26th weeks) | -0.003 (.99) | 1 (7.7) |
| 3rd Quarter (27th-39th weeks) | 0.854 (<.001) | 0 |
| 4th Quarter (40th-52nd weeks) | 0.520 (.07) | 0 |
| United Kingdom |  |  |
| Entire Period (January 21, 2020 to January 20, 2021) | 0.696 (<.001) | 17 (32.7) |
| 1st Quarter (1st-13th weeks) | -0.217 (.48) | 10 (76.9) |
| 2nd Quarter (14th-26th weeks) | 0.524 (.07) | 0 |
| 3rd Quarter (27th-39th weeks) | 0.907 (<.001) | 6 (46.2) |
| 4th Quarter (40th-52nd weeks) | 0.575 (.04) | 1 (7.7) |
| **B. Shared symptoms relative search values** |  |  |
| Australia |  |  |
| Entire Period (January 21, 2020 to January 20, 2021) | -0.118 (.41) | 10 (19.2) |
| 1st Quarter (1st-13th weeks) | 0.463 (.11) | 6 (46.2) |
| 2nd Quarter (14th-26th weeks) | 0.607 (.03) | 3 (23.1) |
| 3rd Quarter (27th-39th weeks) | -0.028 (.93) | 1 (7.7) |
| 4th Quarter (40th-52nd weeks) | 0.486 (.09) | 0 |
| Canada |  |  |
| Entire Period (January 21, 2020 to January 20, 2021) | 0.413 (.002) | 9 (17.3) |
| 1st Quarter (1st-13th weeks) | 0.066 (.83) | 4 (30.8) |
| 2nd Quarter (14th-26th weeks) | 0.318 (.29) | 0 |
| 3rd Quarter (27th-39th weeks) | 0.835 (<.001) | 0 |
| 4th Quarter (40th-52nd weeks) | -0.257 (.40) | 5 (38.5) |
| Ireland |  |  |
| Entire Period (January 21, 2020 to January 20, 2021) | 0.332 (.02) | 17 (32.7) |
| 1st Quarter (1st-13th weeks) | -0.483 (.09) | 6 (46.2) |
| 2nd Quarter (14th-26th weeks) | 0.464 (.11) | 0 |
| 3rd Quarter (27th-39th weeks) | 0.515 (.07) | 5 (38.5) |
| 4th Quarter (40th-52nd weeks) | 0.612 (.03) | 6 (46.2) |
| New Zealand |  |  |
| Entire Period (January 21, 2020 to January 20, 2021) | -0.157 (.27) | 18 (34.6) |
| 1st Quarter (1st-13th weeks) | 0.726 (.005) | 2 (15.4) |
| 2nd Quarter (14th-26th weeks) | 0.471 (.10) | 9 (69.2) |
| 3rd Quarter (27th-39th weeks) | 0.124 (.69) | 7 (53.8) |
| 4th Quarter (40th-52nd weeks) | 0.596 (.03) | 0 |
| United Kingdom |  |  |
| Entire Period (January 21, 2020 to January 20, 2021) | 0.379 (.006) | 19 (36.5) |
| 1st Quarter (1st-13th weeks) | 0.138 (.65) | 8 (61.5) |
| 2nd Quarter (14th-26th weeks) | 0.233 (.44) | 2 (15.4) |
| 3rd Quarter (27th-39th weeks) | 0.739 (.004) | 4 (30.8) |
| 4th Quarter (40th-52nd weeks) | 0.561 (.046) | 5 (38.5) |
